# Development of a Mobile Health Application Based on a Mixed Prenatal Care in the Context of COVID-19 Pandemic

**DOI:** 10.1055/s-0043-1768998

**Published:** 2023-05-24

**Authors:** Rommy Helena Novoa, Luis Meza-Santibañez, Noe Rodríguez-Hilario, Juan Torres-Osorio, Vladimir Jáuregui-Canchari, Xin Huang-Yang, Wilder Eduardo Melgarejo, Juan Carlos Bazo-Alvarez, Walter Ventura

**Affiliations:** 1 Instituto Nacional Materno Perinatal, Lima, Peru; 2 Universidad Nacional Mayor de San Marcos, Lima, Peru; 3 Universidad Peruana Cayetano Heredia, Lima, Peru; 4 University College London, London, United Kingdom; 5 British Medical Hospital, Lima, Peru

**Keywords:** COVID-19, Mobile application, Telemedicine, Prenatal care, Acceptability

## Abstract

**Objective** We describe the development and structure of a novel mobile application in a mixed model of prenatal care, in the context of the COVID-19 pandemic. Furthermore, we assess the acceptability of this mobile app in a cohort of patients.

**Methods** First, we introduced a mixed model of prenatal care; second, we developed a comprehensive, computer-based clinical record to support our system. Lastly, we built a novel mobile app as a tool for prenatal care. We used Flutter Software version 2.2 to build the app for Android and iOS smartphones. A cross-sectional study was carried out to assess the acceptability of the app.

**Results** A mobile app was also built with the main attribute of being connected in real-time with the computer-based clinical records. The app screens detail information about activities programmed and developed in the prenatal care according to gestational age. A downloadable maternity book is available and some screens show warning signs and symptoms of pregnancy. The acceptability assessment was mostly rated positively regarding the characteristics of the mobile app, by 50 patients.

**Conclusion** This novel mobile app was developed as a tool among pregnant patients to increase the information available about their pregnancies in the provision of a mixed model of prenatal care in the context of the COVID-19 pandemic. It was fully customized to the needs of our users following the local protocols. The introduction of this novel mobile app was highly accepted by the patients.

## Introduction

Perinatal care is a public health strategy that has been thought to be one of the most effective means of reducing unfavorable perinatal outcomes. 1 However, the restrictions such as immobilization and lockdown to limit the spread of COVID-19 caused the health services to the outpatient clinics to be interrupted associated with an increased frequency of adverse maternal and perinatal outcomes. 2 3 Given this scenario and the need to assure the continuity of the provision of prenatal care, we introduced a new model of mixed prenatal with in-person visits and a teleconsultation program. 4 5 Telemedicine has been proven to be useful in the care of pregnant women in different scenarios to provide health outcomes comparable to the traditional methods of care. 6 7 8 9 Therefore, the major role of integrating electronic health technology into prenatal care is to provide broader healthcare in diverse manners and to create a lot of opportunities for patients and health providers.

Studies of women's general assessments of what they perceive as important aspects of antenatal care reported that sufficient information and explanation were so important. 10 11 We believe it is imperative to allow patients actively participation in their pregnancy care. The availability of more information about her health status and that of her baby allows the pregnant woman to be involved in the entire care process. This achieves feedback between the pregnant woman and the doctor that will improve maternal and perinatal outcomes. Mobile technology, a modality of telehealth, has been reported as a useful and reliable tool for monitoring clinical factors and treatment in different health conditions. 12 Thus, the introduction of new technology as a mobile application with data regarding their pregnancy could help our patients with this objective.

In this study, we describe the development and structure of a novel mobile application in mixed prenatal care in the context of the COVID-19 pandemic and tested its acceptability in a cohort of patients.

## Methods

We introduced telemedicine and mobile technology considering recommendations from frameworks on the development of health-related interactive systems. 13 14 This study was part of a larger institutional study on COVID-19 (reference number: 063-2020-DG-N°20-OEAIDE/INMP) approved by the local institutional ethics board (reference number: 019-2020-CIEI/INMP).

### Insights

We grouped a multidisciplinary team including TI people, OB/GYN doctors, statisticians, and external consultants supported by the hospital managers and the Ministry of Health to build a new model of prenatal care adapted to the new scenery of the COVID-19 pandemic. We followed 3 steps. First, we introduced a mixed model of prenatal care based on international recommendations, 15 16 17 which essentially considers a reduced number of in-person visits and some virtual phone consultations. A detailed description of this new care model was published in a previous article. 5 Second, we developed a comprehensive clinical computer-based patient record (Integrated Hospital Management System, SISGALEN PLUS®, INMP-MINSA, Peru), built on our standard model of prenatal care previously established in agreement with CLAP recommendations and other current international guidelines. 8 17 18 Third, we developed a mobile application to allow patients to actively participate in their pregnancy care.

### Design and Build of the Mobile Application and Prototype Characteristics

In this context, our institution developed a mobile application to help provide comprehensive and personalized prenatal care. This technology was designed and developed by OB/GYN doctors and computer engineers based on the novel mixed model of prenatal care with continuous feedback from patients. We used Flutter Software version 2.2 to build the mobile application for Android and iOS smartphones. It took 6 months from design and construction to be introduced into the Google store. It will be soon introduced into the Apple store.

### Acceptability Assessment

We assessed the app's acceptability using an instrument for evaluating a telehealth program proposed by Portz et al. 19 This survey is divided into 2 sections. The first one includes 8 questions measured on a 5-point Likert scale ranging from “extremely disagree” to “extremely agree,” and the second section includes 3 open-ended questions. According to the technology acceptance model (TAM), our survey was applied to understand patients' adoption of new mobile application. 20 21 The 4 TAM constructs applied were 1) *perception of the app's usefulness* (1 question), 2) *perception of the app's ease of use* (5 questions), 3) *attitudes* about the app (2 questions), and 4) *intentions to use* the app (1 open question) ( Supplemental 1 ). We collected survey data in a cohort of patients with access to mixed prenatal care who were invited to participate in the study in a non-random fashion. Patients received comprehensive information on how to download and run the app, before the acceptability survey. Demographic characteristics were collected directly from the patients and electronic records. The acceptability was a paper questionnaire, carried out during the last in-person visit. Answers could be clarified at the time of the survey by the researchers.

**Chart 1:** Demographic and maternal characteristics (n = 50)

	n	%
Mean maternal age, years (range)	30.0 (17.8–41.2)	
Mean gestational age at first contact with the mobile app, weeks (range)	27.8 (12.1–37.1)	
Human Development Index		
Stratum I	2	4.0
Stratum II	22	44.0
Stratum III	21	42.0
Stratum IV	5	10.0
Insurance modality		
National health insurance	43	86.0
Not national health insurance	7	14.0
Education level		
Primary	3	6.0
High school	36	72.0
Technical or University	11	22.0
Nulliparous	15	30.0
Risk factors		
Obesity (BMI≥30)*	29	58.0
Previous cesarean section	20	40.0
Mother Rh negative	11	22.0
Fetus with structural abnormalities	10	20.0
Diabetes	7	14.0
Hypothyroidism/Hyperthyroidism	6	12.0
History of hypertension/preeclampsia	6	12.0
Anemia	5	10.0
Multiple pregnancy	3	6.0
Placenta previa	2	4.0
Asthma	2	4.0
Short interpregnancy interval	1	2.0
Mother with HIV** infection	1	2.0
Previous perinatal death	1	2.0
Others	6	12.0
Operating system used by the patients		
iOS	2	4.0
Android	48	96.0

*BMI: Body mass index

**HIV: Human immunodeficiency virus

We performed a descriptive analysis using MS Excel 2013. Results from the acceptability survey were summarized for each point of the Likert scale and represented in a stacked bar chart for the 9-item questions. 22 The open-ended responses were analyzed using magnitude coding, which quantifies participants' answers, highlighting the most frequent comments. Statistical analysis was performed using Stata Statistical Software 14.0 (Stata Corp. 2015, College Station, TX, USA).

## Results

### Design and Build of the Mobile Application and Prototype Characteristics

**Fig. 1:**
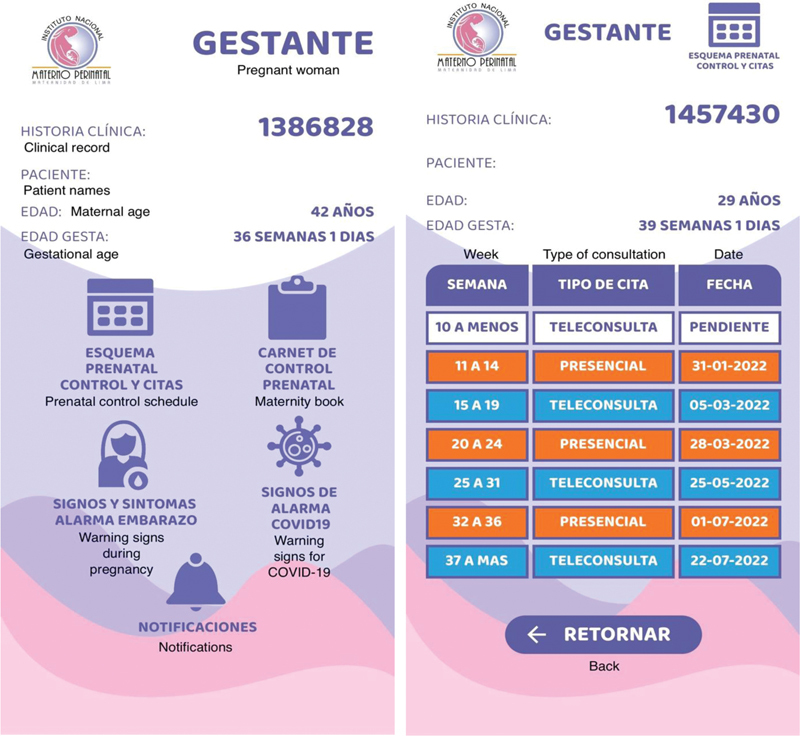
Prototype's main screens and prenatal control schedule

**Fig. 2:**
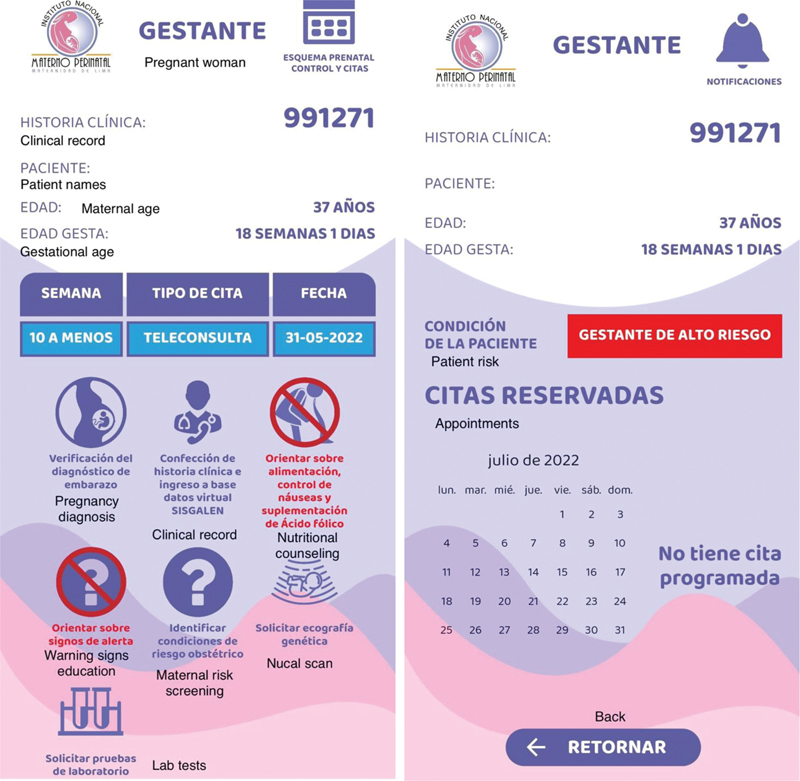
Scheduled tasks for the corresponding gestational age and notifications screen

We built a mobile application with the main attribute of being connected in real time with the clinical computer-based record. The system was structured on a distributed architecture of microservices, then they are consumed by an app that is structured with a hybrid development through an “API Gateway”. This allows great versatility to be able to deploy it on Android and IOS cell phones. This mobile application can be delivered exclusively to patients with an electronic clinical record, accessed with a username defined by the national ID, and a protected password. The built app's main screens are shown in Figures 1 and 2 , and additional screens are provided in Supplementals . The first screen of visualization ( Figure 1 ) shows the name, past medical history, and age of the patient, as well as the current gestational age.

It contains 5 options listed as follows: 1) *prenatal control schedule* including all the tasks for the corresponding gestational age, 2) *prenatal card* with the relevant clinical data and lab tests, 3) *information about the alarm signs* of the current pregnancy, 4) *list of symptoms* of COVID-19 disease, and 5) *notifications* about omitted tasks. By clicking on the prenatal control schedule, patients can visualize the prenatal protocol care summarized in 6 appointments ( Figure 1 ), each one corresponding to a specific period of pregnancy carried out either by virtual or in-person consultation. In addition, the gestational age of the patient at the time of the appointment can be visualized. Each completed visit is marked in orange, and the subsequent appointments are displayed in white ( Figure 2 ).

By clicking on each appointment, a screen is displayed with all the scheduled tasks for the corresponding gestational age ( Figure 2 ), such as clinical evaluation, obstetric ultrasound scan, lab tests, provision of medications, Pap smear test, vaccination, family planning counseling, and psychoprophylaxis. Tasks not carried out will be marked in red. The option of the maternity book enables the user to download a printable version of the updated information about clinical history and lab test results. Additionally, patients have access to information about the alarm signs of pregnancy, COVID-19 disease ( Supplemental Figures ), and notifications about omitted activities and scheduled appointments ( Figure 2 ). We built several previous versions, which were modified according to patient feedback. A final version was tested for connectivity with the clinical computer-based record.

### Assessment of the Acceptability

Fifty patients were surveyed in the study period in our outpatient clinic. All gave written consent to participate in the survey. Chart 1 details the characteristics of the study population. The mean maternal age was 30 years, and the mean gestational age at first contact with the mobile application was 27.8 weeks. Forty-three (86%) patients had national health insurance, and 11 patients (22%) reported having access to education beyond high school. All pregnant women had at least one risk factor, including overweight and obesity (n = 29, 58%) and previous cesarean sections (n = 20, 40%).

**Fig. 3:**
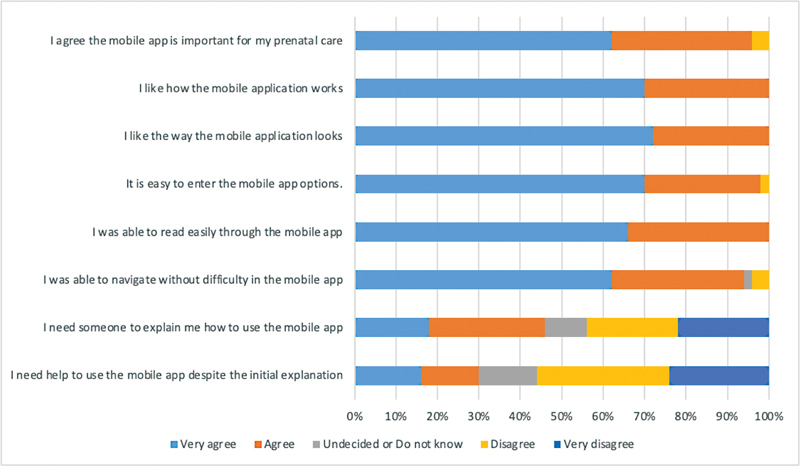
Acceptability survey of the mobile app among pregnant women (n = 50)

Regarding the type of mobile phone, 48 (96%) reported using the Android operating system. Figure 3 shows the patients' acceptance of the mobile application. The 4 constructs of acceptability yielded the following results: 1) *perception of usefulness* : 96% (n = 48) agree that the mobile app is essential and will help with their prenatal care; 2) *perception of ease of use* : more than 94% of patients were able to enter, read, and navigate through the mobile app, but 46% (n = 23) still needed some orientation and help to use the application; 3) *attitudes about the app* : all patients were pleased with how the application works and looks; and 4) *intention to use* : 94% (n = 47) of patients reported the intention to use the app again. Regarding the open question asking “What do you like the most about the application?” 62% of patients like the information about their pregnancy, visibility of lab test results, and subsequent appointment, and 22% of women like it because it is easy to use. Only 20% of patients gave some suggestions to improve the application. Six patients recommended that the app be available to all pregnant women, 2 of them suggested including notifications a day before the appointment, and 1 patient suggested the possibility to contact doctors online anytime.

## Discussion

This study details the development and structure of a novel mobile application in a mixed model of prenatal care in the context of the COVID-19 pandemic. The assessment of the app's acceptability was mostly positive.

There are several mobile applications commercially available dedicated to some aspect of pregnancy care, 23 however, not all have an impact on improving maternal health. Cawley et al. 24 reported using a mobile application based on information tips to enhance healthy behaviors among pregnant women but with no impact on clinical health outcomes. Innovative solutions are recommended to closely manage, monitor, and empower pregnant women to actively participate in the management of their pregnancy. 25 26 A mobile health app that targets pregnant women may facilitate the integration of prenatal care into other aspects of their family and professional life. Thus, women who are highly engaged with their healthcare decisions during pregnancy might be more receptive to educational programs and recommendations. 27 Our new mobile application allows patients to access some aspects of the clinical record, enhancing personalized care. In our scenario, patients have a prenatal card as proof of compliance with the traditional prenatal care protocol summarizing the main achieved activities. Therefore, the new app described here still allows patients to view an updated printable card anytime and anywhere. Thus, if the app can communicate prenatal care information and basic alarm signs of pregnancy, the in-person visits may allow for more individualized discussion. Ultimately, health managers and providers must ensure the privacy and security of patients' information when using telemedicine. 28 Therefore, we adhered to a strict security protocol when developing this app, allowing access exclusively to patients with a valid ID card and an encrypted password.

This mobile application was tested with a considerable number of high-risk pregnant women and showed an acceptable perception regarding the characteristics of the application in 3 of the 4 constructs evaluated: perception of usefulness, attitudes toward the app, and intention to use. However, some aspects of the ease of using the app among the patients could dampen the usability of this new technology. These difficulties probably occur due to the inexperience of patients with mobile applications that provide health care information despite 94% of them having an educational level higher than high school. Several improvements to the app should be incorporated for future use, and instructions for use should be provided to future users. Because the inexperience was associated with a need for assistance to use the app, instructions, and support from health providers will be important to engage pregnant patients with the app. Final changes to our app should also include improvements in mobile platform capability to support the number of patients in prenatal care.

## Conclusion

To our knowledge, this is the first study to introduce a mobile application among pregnant patients during the COVID-19 pandemic scenery as a tool to increase the information available about their pregnancies in a mixed prenatal care program in a low-resource country. Our results provide evidence of the high acceptability of this mobile application among users, which is an essential step to massifying this tool in routine prenatal care. However, further studies are needed to test the impact of this novel application among perinatal outcomes.
