# Supplementary material for: Development of a Mobile Health Application Based on a Mixed Prenatal Care in the Context of COVID-19 Pandemic
Source: Rev Bras Ginecol Obstet. 2023 May 24;45(4):179–85. doi: 10.1055/s-0043-1768998 (PMC10208727; doi:10.1055/s-0043-1768998)
Supplement: Supplementary file 1 — Supplementary Material [file 10-1055-s-0043-1768998-s220258.pdf]

Supplemental 1 Aspects of acceptability survey of the mobile app among pregnant women

Acceptability aspects and details
**Perceived usefulness**
I like how the mobile application works
I agree the mobile application is important for my prenatal care
**Behavioural intentions (Attitude)**
I like the way the mobile application looks
It is easy to enter the mobile application options.
I need someone to explain me how to use the mobile application
I need help to use the mobile application despite the initial explanation
**Perceived ease of use**
I was able to read easily through the mobile application
I was able to navigate without difficulty in the mobile application
**Intention to use**
Would you use the mobile application again in the future?

Scale: 1 = Very disagree, 2 = Disagree, 3 = Undecided, 4 = Agree, 5 = Very agree.
**Scale was applied to first three constructs.

 © 2023. Federação Brasileira de Ginecologia e Obstetrícia. All rights reserved.

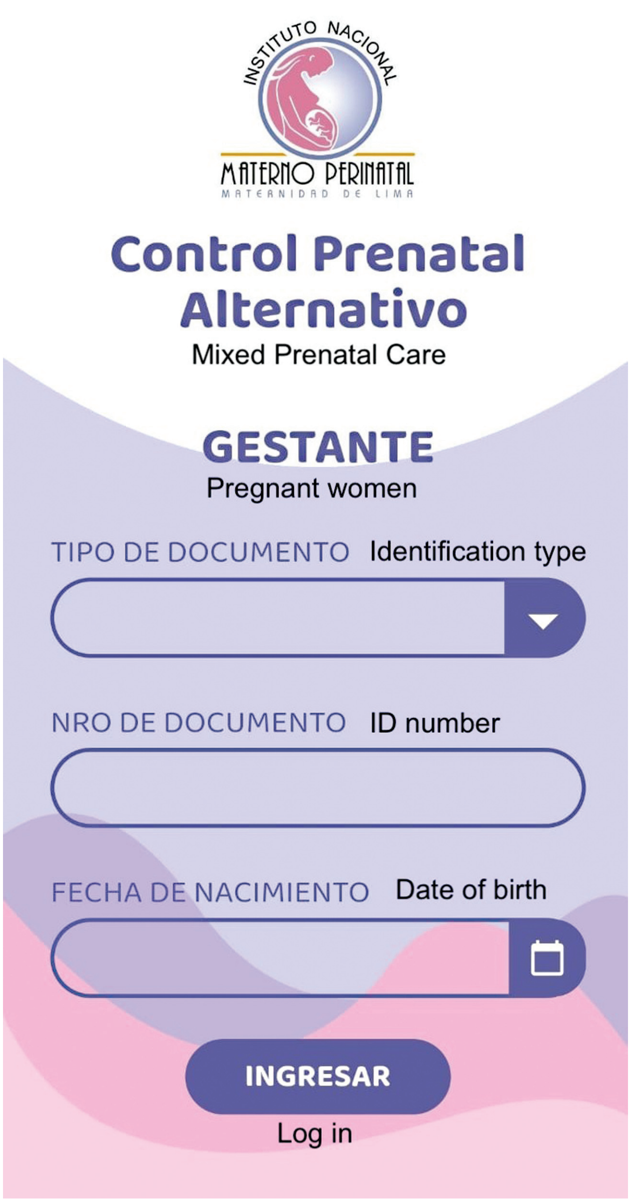

Supplemental Figure S1 Screens of the mobile app: ID screen

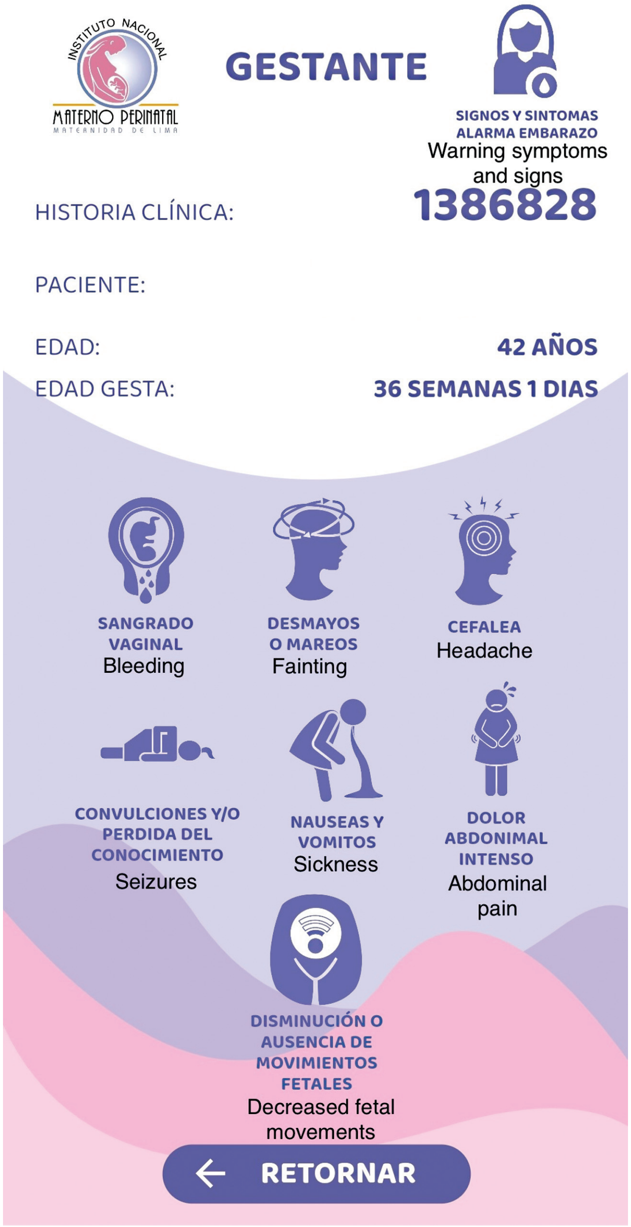

Supplemental Figure S2 Screens of the mobile app: Alarm signs of the current pregnancy

 © 2023. Federação Brasileira de Ginecologia e Obstetrícia. All rights reserved.

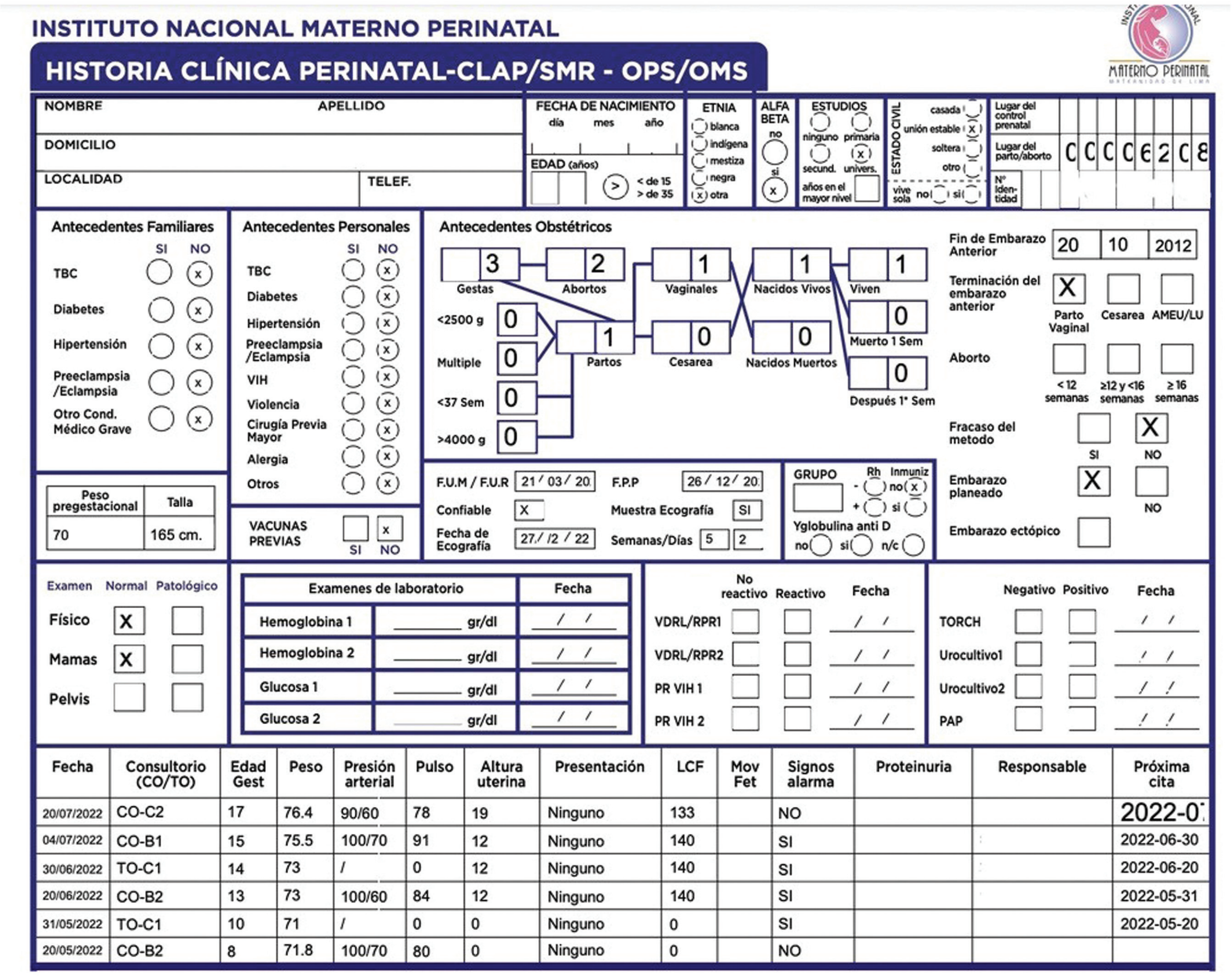

INSTITUTO NACIONAL MATERNO PERINATAL

HISTORIA CLÍNICA PERINATAL-CLAP/SMR - OPS/OMS

MATERNO PERINATAL

NOMBRE APELLIDO

DOMICILIO

LOCALIDAD TELEF.

FECHA DE NACIMIENTO día mes año

EDAD (años) < de 15 > de 35

ETNIA blanca indígena mestiza negra (x) otra

ALFA BETA no si (x)

ESTUDIOS ninguno primaria secund. univers. (x) años en el mayor nivel

ESTADO CIVIL casada unión estable (x) soltera otro vive sola no si

Lugar del control prenatal

Lugar del parto/aborto C C C C E 2 C 8

Nº Identidad

Antecedentes Familiares (SI / NO): TBC (x NO), Diabetes (x NO), Hipertensión (x NO), Preeclampsia /Eclampsia (x NO), Otro Cond. Médico Grave (x NO)

Antecedentes Personales (SI / NO): TBC (x NO), Diabetes (x NO), Hipertensión (x NO), Preeclampsia /Eclampsia (x NO), VIH (x NO), Violencia (x NO), Cirugía Previa Mayor (x NO), Alergia (x NO), Otros (x NO)

Antecedentes Obstétricos: Gestas 3; Abortos 2; Vaginales 1; Nacidos Vivos 1; Viven 1; <2500 g 0; Multiple 0; <37 Sem 0; >4000 g 0; Partos 1; Cesarea 0; Nacidos Muertos 0; Muerto 1 Sem 0; Después 1° Sem 0

Fin de Embarazo Anterior 20 10 2012

Terminación del embarazo anterior: Parto Vaginal X; Cesarea; AMEU/LU

Aborto: < 12 semanas; ≥12 y <16 semanas; ≥ 16 semanas

Fracaso del metodo: SI; NO X

Embarazo planeado: SI X; NO

Embarazo ectópico

Peso pregestacional 70; Talla 165 cm.

VACUNAS PREVIAS SI; NO x

F.U.M / F.U.R 21 / 03 / 20; F.P.P 26 / 12 / 20

Confiable X; Muestra Ecografía SI

Fecha de Ecografía 27 / 12 / 22; Semanas/Días 5 2

GRUPO; Rh - +; Inmuniz no (x) si

Yglobulina anti D no si n/c

Examen	Normal	Patológico
Físico	X	
Mamas	X	
Pelvis		

Examenes de laboratorio		Fecha
Hemoglobina 1	gr/dl	/ /
Hemoglobina 2	gr/dl	/ /
Glucosa 1	gr/dl	/ /
Glucosa 2	gr/dl	/ /

	No reactivo	Reactivo	Fecha
VDRL/RPR1			/ /
VDRL/RPR2			/ /
PR VIH 1			/ /
PR VIH 2			/ /

	Negativo	Positivo	Fecha
TORCH			/ /
Urocultivo1			/ /
Urocultivo2			/ /
PAP			/ /

Fecha	Consultorio (CO/TO)	Edad Gest	Peso	Presión arterial	Pulso	Altura uterina	Presentación	LCF	Mov Fet	Signos alarma	Proteinuria	Responsable	Próxima cita
20/07/2022	CO-C2	17	76.4	90/60	78	19	Ninguno	133		NO			2022-0[illegible]
04/07/2022	CO-B1	15	75.5	100/70	91	12	Ninguno	140		SI			2022-06-30
30/06/2022	TO-C1	14	73	/	0	12	Ninguno	140		SI			2022-06-20
20/06/2022	CO-B2	13	73	100/60	84	12	Ninguno	140		SI			2022-05-31
31/05/2022	TO-C1	10	71	/	0	0	Ninguno	0		SI			2022-05-20
20/05/2022	CO-B2	8	71.8	100/70	80	0	Ninguno	0		NO			

Supplemental Figure S3 Maternity book in downloadable printable version

 © 2023. Federação Brasileira de Ginecologia e Obstetrícia. All rights reserved.
